# Maternal Smoking, Vaping and Infant Sleep Practices in Sudden Unexpected Death in Infancy: A New Zealand Case Series

**DOI:** 10.1111/jpc.70352

**Published:** 2026-03-15

**Authors:** Edwin A. Mitchell, Stephanie Cowan, Barry J. Taylor

**Affiliations:** ^1^ Department of Paediatrics, Child and Youth Health The University of Auckland Auckland New Zealand; ^2^ Change for our Children Christchurch New Zealand; ^3^ Department of Paediatrics and Child Health University of Otago Dunedin New Zealand

**Keywords:** bed sharing, ethnicity, infant mortality, smoking, sudden unexpected death in infancy, vaping

## Abstract

**Background:**

New Zealand's sudden unexpected death in infancy (SUDI) rates remain high, and ethnic disparities appear to be increasing. This study describes the characteristics and circumstances of these deaths.

**Methods:**

Coronial data identified 101 SUDI cases in 2022–2023. Sociodemographic factors, sleep environment, and maternal smoking and/or vaping were analysed and compared with previous years.

**Results:**

The SUDI rate was 0.87/1000 live births. Rates among Māori (1.97/1000) and Pacific (0.91/1000) infants were higher than those among Europeans (0.17/1000). 72.3% of cases occurred while direct bed sharing. Maternal smoking was reported in 51.5% of cases, vaping in 30.3%, and smoking and/or vaping in 72.7%. Vaping was more likely in bed‐sharing than non‐bed‐sharing deaths (*p* = 0.003). Compared with New Zealand studies from 1987 to 1990 and 2012 to 2015, current cases show increasing proportions of Māori and Pacific infants, older mothers, back sleeping and direct bed sharing.

**Conclusions:**

SUDI rates remain unacceptably high with widening ethnic disparities. The proportion of deaths associated with direct bed sharing is striking and is increasing. Vaping appears to be an emerging SUDI risk factor, especially when combined with direct bed sharing. Further studies examining the association of vaping with SUDI are urgently needed.

## Key Messages

- What is already known on this topic
  ○ Maternal smoking in pregnancy and direct bed sharing are major risk factors for sudden unexpected death in infancy (SUDI), especially when they occur together.
  ○ In New Zealand, smoking in pregnancy has decreased, but paradoxically, SUDI rates remain unchanged.
- What this study adds
  ○ Vaping is an emerging SUDI risk, especially when combined with direct bed sharing and may help explain this paradox.
  ○ Further studies examining the association of vaping with SUDI are urgently needed.

## 1 Introduction

Sudden unexpected death in infancy (SUDI) refers to the sudden death of an infant under 1 year of age, occurring during sleep. It includes sudden infant death syndrome (SIDS) and other deaths where a possible cause, such as accidental asphyxia, may be identified. Although ‘Back to Sleep’ campaigns advising the back sleeping position greatly reduced infant mortality, SUDI remains a major concern in New Zealand. Established modifiable risks include prone sleeping position, maternal smoking, lack of breastfeeding, and bed sharing. A strong interaction between maternal smoking and bed sharing, first shown in New Zealand [1] and confirmed elsewhere [2, 3, 4], greatly increases risk.

New Zealand has implemented various ‘safe‐sleep’ initiatives, including the targeted provision of in‐bed sleepers like Wahakura and Pēpi‐Pod, which were associated with a fall in post‐perinatal mortality [5]. However, despite these efforts, New Zealand's SUDI rates have plateaued.

This report aims to describe the characteristics and circumstances of SUDI deaths occurring in New Zealand during 2022 and 2023, drawing attention to prevailing risk factors and emerging concerns.

## 2 Methods

This analysis of SUDI cases occurring in 2022 and 2023 was conducted using coronial data. Cases were assigned as SUDI by expert review (E.A.M., with unclear cases reviewed by B.J.T.) if the infant death was sudden and unexpected, occurred when the infant (less than 1 year of age) was thought to be asleep, and a postmortem examination did not identify an alternative explanation for the death.

Detailed sociodemographic information was available from the Coronial Infant Death file, which included a short report on the circumstances for all (*n* = 101) cases. Detailed reports by the SUDI Liaison were available for 66 out of 101 cases (65.3%). SUDI Liaisons are health‐trained investigators equivalent to medical investigators in the United States who use a standardised approach and structured questions for reporting to the coroner.

Information extracted included infant sex, ethnicity, age at death, sleeping surface, bed‐sharing status, position placed to sleep, dummy (pacifier) use, maternal smoking (in pregnancy or after birth), maternal vaping (in pregnancy or after birth), father's smoking status, other household members' smoking, breastfeeding status, and the presence of an in‐bed sleeper in the house. Direct bed sharing was defined as the baby sharing the same sleep surface with another person at the time of death. Data were extracted into an Excel spreadsheet, and descriptive data were presented. Denominator data for mortality rates were obtained from Stats NZ Infoshare.

For comparison of smoking/vaping rates in the bed‐sharing group with those in the non‐bed‐sharing group, chi‐squared statistics or Fisher's exact test (when a cell was < 5) and Cramer's *V* were calculated. Cramer's *V* provides a measure of the strength of an association. Greater than 0.3–0.5 indicates a moderately strong association.

Case data from two previously reported New Zealand case–control studies were extracted and compared with the current cases. In testing for trend over time, either the Cochran–Armitage test for trend of binary outcomes was used, or for ordered multi‐category outcomes (birthweight, mother's age), the Mantel linear‐by‐linear association test was used.

Ethics approval for this study was granted by the Northern A Health and Disability Ethics Committee (17/NTA/245).

## 3 Results

A total of 101 SUDI cases occurred in New Zealand during 2022 and 2023, resulting in an overall SUDI mortality rate of 0.87 per 1000 live births. There were marked ethnic disparities: Māori 1.97/1000, Pacific 0.91/1000, Asian 0.07/1000 and European 0.17/1000. The majority of these deaths occurred within the first 3 months of life, with specific infant demographics and birth characteristics detailed in Tables 1 and 2.

**TABLE 1:** Sociodemographic factors, pregnancy and infant care practices (*n* = 101).

	*N*	%
Ethnicity		
Māori	69	68.3
Pacific	18	17.8
Asian	2	2.0
European	12	11.9
Sex of infant		
Male	62	61.4
Female	39	38.6
Age of infant at death (completed weeks)		
< 13	64	63.4
13–25	22	21.8
26–38	9	8.9
39–51	6	5.9
Season		
December–February	19	18.8
March–May	23	22.8
June–August	33	32.7
September–November	26	25.7
Bed‐sharing at the time of death		
Yes	73	72.3
No	28	27.7
Sleeping surface at time of death		
Bed/mattress	68	67.3
Bassinet/cot	14	13.9
Portable cot/Porta cot	3	3.0
Pēpi‐Pod/wahakura	4	4.0
Sofa/recliner chair	7	6.9
Bean bag	1	1.0
Miscellaneous	4	4.0

**TABLE 2:** Data from SUDI Liaison reports (*n* = 66).

	*N*	%
Preterm birth < 37 weeks' gestation		
Yes	19	28.8
No	47	71.2
Birthweight (g)		
< 2500	20	30.8
2500–2999	15	23.1
3000–3499	11	16.9
3500–3999	15	23.1
4000+	4	6.2
UK	1	
Position placed to sleep		
Back	48	77.4
Side	9	14.5
Front	5	8.1
DK/unsure	4	
Dummy/pacifier used last sleep		
Yes	24	37.5
No	40	62.5
DK	2	
Mother smoked in pregnancy		
Yes	34	51.5
No	32	48.5
Mother smoked after birth		
Yes	32	49.2
No	33	50.8
DK	1	
Mother vaped in pregnancy		
Yes	20	30.3
No	46	69.7
Mother vaped after birth		
Yes	24	36.9
No	41	63.1
DK	1	
Father smoked after birth		
Yes	30	57.7
No	22	42.3
DK	6	
NA	8	
Breastfeeding ever		
Yes	60	90.9
No	6	9.1
In‐bed sleeper in house		
Pēpi‐Pod	15	22.7
Wahakura	14	21.2
Pēpi‐Pod and wahakura	2	3.0
Yes, type not specified	5	7.6
No	30^a^	45.5

^a^ Eight were offered an in‐bed sleeper but declined, one was supplied with a bassinet.

A striking finding was that 72.3% of deaths occurred while the infant was directly bed sharing (Table 1). While infants were most frequently found on a standard bed or mattress, a variety of other surfaces were recorded, ranging from sofas to portable sleepers; a comprehensive breakdown of these sleep environments is provided in Table 1.

Despite long‐standing ‘Back to Sleep’ campaigns, a notable minority of infants were still being placed in non‐supine positions, though the majority were placed on their backs.

Maternal smoking and vaping were prevalent in these data, with nearly three‐quarters of all cases involving maternal smoking, vaping or both (Table 3). Vaping has emerged as a particularly frequent factor, with 40.9% of mothers reporting use during pregnancy and/or after birth. Crucially, the data reveal a significant statistical association between these habits and sleep practices: infants who died while bed sharing were much more likely to have been exposed to maternal smoking or vaping compared to those who were not bed sharing.

**TABLE 3:** Association of maternal smoking and vaping with bed sharing status.

	Bed sharing (*n* = 46)	No bed sharing (*n* = 20)	Statistical test	*p*	Cramér's *V*
Any smoking or vaping	39 (84.8%)	9 (45.0%)	*χ* ^2^ = 11.13	< 0.001	0.41
Neither smoking nor vaping	7 (15.2%)	11 (55.0%)	Reference		
Any smoking	28 (60.9%)	8 (40.0%)	*χ* ^2^ = 7.96	0.005	0.38
Any vaping	23 (50.0%)	4 (20.0%)	Fisher's exact	0.003	0.48

*Note:* Data based on the 66 cases with complete SUDI Liaison reports. Percentages refer to the proportion within the bed‐sharing or non‐bed‐sharing group.

In‐bed sleepers (Pēpi‐Pod/wahakura) in the home were present for 36 (54.5%) families but were used at the time of death by only 4. A total of 30 had no in‐bed sleeper, although 8 of these were offered 1 but declined (1 was supplied with a bassinet).

There were several significant shifts in risk factors compared with historical New Zealand data from 1987 to 1990 [1, 6] and 2012 to 2015 [7] (Table 4), including a substantial increase in the proportion of Māori and Pacific cases, alongside a rising trend in maternal age and a continued increase in the frequency of bed sharing. Conversely, positive trends include a significant decrease in prone sleeping and a recent decline in maternal smoking during pregnancy. Further longitudinal comparisons regarding birthweight, breastfeeding, and sleep positions are detailed in Table 4.

**TABLE 4:** Comparison of characteristics and infant care practices among SUDI cases in the NZ Cot Death Study (1987–1990), Nationwide SUDI Case–Control Study (2012–2015), and this SUDI case series (2022 and 2023).

	NZ cot death study		SUDI case control study		SUDI case series				
	1 Nov 1987–31 Oct 1990		1 Mar 2012–28 Feb 2015		2022–2023				
Mortality rate/1000 live births	3.53		0.76		0.87				
	*N*	%	*N*	%	*N*	%	Trend	Test	*p*
Ethnicity									
Māori	229	48.8	63	49.6	69	68.3	↑	*Z* = 3.159	0.002
Pacific	30	6.4	19	15	18	17.8	↑	*Z* = 4.091	< 0.001
Other	210	44.8	45	35.4	14	13.9	↓	*Z* = −5.720	< 0.001
Sex of infant									
Male	284	61.1	77	57.9	62	61.4	↔	*Z* = −0.182	0.856
Female	181	38.9	56	42.1	39	38.6			
Birthweight (g)									
< 2500	82	17.6	15	12.6	20	30.8	↔	*χ* ^2^ = 1.378	0.240
2500–2999	111	23.9	29	24.4	15	23.1			
3000–3499	135	29.0	42	35.3	11	16.9			
3500+	137	29.5	33	27.7	19	29.2			
Mother's age									
< 20	87	18.7	26	21.1	8	12.1			
20–24	157	33.8	45	36.6	17	25.8			
25–29	139	29.9	27	22.0	16	24.2			
30+	82	17.6	25	20.3	25	37.9	↑	*χ* ^2^ = 1.378	0.019
Mother smoked while pregnant									
Yes	285	64.8	92	74.2	34	51.5	↔	*Z* = −0.873	0.382
No	155	35.2	32	25.8	32	48.5			
Breastfeeding ever									
Yes	342	87.2	115	89.8	60	90.9	↔	*Z* = 1.047	0.295
No	50	12.8	13	10.2	6	9.1			
Position placed to sleep									
Back	18	4.7	83	65.9	48	77.4	↑	*Z* = 16.023	< 0.001
Side	120	30.9	31	24.6	9	14.5			
Front	250	64.4	12	9.5	5	8.1			
Bed‐sharing									
Yes	94	24.0	73	57.5	73	72.3	↑	*Z* = 9.945	< 0.001
No	297	76.0	54	42.5	28	27.7			

## 4 Discussion

Analysis of SUDI deaths in New Zealand during 2022 and 2023 shows a persistently high rate (0.87/1000 live births), well above Australia (0.32/1000 live births in 2023) and Northern Europe (0.1/1000) [8]. Despite decreases in NZ of infant prone sleeping and maternal smoking in pregnancy, SUDI mortality has not declined in the last decade. This suggests that new or increasing risk factors are contributing, or existing ones are becoming more prevalent and/or impactful.

Persistent and widening ethnic disparities are evident, with Māori infants accounting for 68.3% of deaths. This aligns with earlier reports that Māori infants are disproportionately exposed to recognised SUDI risks—especially smoking and direct bed sharing—as well as socio‐economic and demographic factors. Although the risk associated with smoking and bed sharing is similar across ethnicities, their higher prevalence among Māori results in a greater SUDI burden [9].

The most striking finding is the high proportion of deaths (72.3%) occurring during direct bed sharing—a marked increase from 24.0% in 1987–1990 and 57.5% in 2012–2015. This proportion exceeds UK studies (32% and 36%) [4, 10] and the United States (59.5%) [11]. Evidence suggests that bed sharing increases the risk of SUDI, even without smoking or other hazards, for infants under 3 months, albeit by a small amount [2]. Risk is greatly amplified when combined with smoking. Add maternal alcohol consumption and/or drug use [1, 4, 10, 12], and the risk escalates to alarming levels. Current population data for bed‐sharing prevalence are limited, but records for the same period from Whānau Āwhina Plunket (the major provider of well‐child services) show 18.4% of parents reported bed sharing at 6 weeks [13], far below the 72.3% in this series. This implicates bed sharing as a major contributor to the plateau in SUDI rates. A previous meta‐analysis estimated that about 88% of deaths during bed sharing were attributable to it, assuming causality [2].

Encouragingly, several trends are positive. Fewer deaths now occur among young mothers, and prone or side sleeping has declined, reflecting successful safe‐sleep campaigns. Breastfeeding rates among cases remain high, a recognised protective factor against SIDS. Also, maternal smoking at 2 weeks postnatal has fallen markedly from 13.7% in 2009 to 7.3% in 2021 [14].

The paradox of stable SUDI rates despite positive changes suggests new challenges. Maternal smoking and vaping clustered strongly with bed sharing. The use of Cramer's *V* highlights a substantial overall association without implying causality. These findings should be interpreted strictly as within‐case associations, reflecting exposure co‐occurrence among SUDI cases rather than estimates of population risk. Nonetheless, the consistency and strength of the observed associations support the hypothesis that smoking and vaping are markers of unsafe‐sleep contexts, including bed sharing and reinforce public health messages advising against bed sharing in the presence of any nicotine exposure.

Vaping is widely promoted as a harm reduction for those who smoke, including in pregnancy. A 2015 review reported it was ‘95% less harmful than cigarettes’ [15], but pregnancy was not addressed. Public education endorsed by the Royal College of Midwives (RCM) in 2019 stated that nicotine alone is relatively harmless and reiterated this in 2022 [16]. Yet nicotine crosses the placenta, reduces foetal blood flow and lowers birthweight [17]. We suspect that vaping may also produce the same infant ‘arousal defect’ seen with tobacco exposure [18]. Public‐health messaging should urgently reflect these risks.

A parallel exists with snus (and snuff), a smokeless tobacco high in nicotine and other chemicals, absorbed through the oral mucosa. Cohort studies in Sweden and Norway link snus to reduced birthweight, increased preterm births, stillbirth, post‐neonatal and SUDI mortality and reduced infant lung function [19, 20, 21]. The risks were equivalent to moderate smoking. Awareness of the dangers of snus is high in Scandinavia, and most pregnant users stop before their first antenatal check [22]. In Sweden, the emphasis of public health education has shifted from tobacco smoking to nicotine in all its forms, including vaping [23, 24].

In 2023–2024, 11.1% of New Zealand adults reported daily vaping, with higher prevalence among Māori (28.8%), Pacific peoples (21.5%) and adults aged 18–24 years (26.5%) [25]. By contrast, in Australia, daily vaping was 6.7% among those aged 18–44 and 1.6% among people aged 45 years and older [26]. Australia's lower rates may reflect stricter regulation, including a 2021 prescription requirement for nicotine e‐cigarettes and, from 2024, limiting sales to pharmacies for smoking cessation only [27]. In New Zealand, several measures from the 2022 Smokefree Action Plan—such as the planned reduction in retail outlets, a very low‐nicotine product standard and the proposed ‘smokefree generation’ policy—were recently repealed, potentially slowing progress towards reducing tobacco‐related harm. Current vaping regulation remains comparatively narrow, focusing mainly on penalties for sales to minors and the proximity of specialist vaping stores to early childhood education centres.

As shown in Table 2, 54% of cases had an in‐bed sleeper in the home at the time of death, higher than supply rates reported for 2019–2021 [22]. As most infants older than 13 weeks will have outgrown in‐bed sleepers, a focused look at those under 13 weeks shows that 42 of 64 (65.6%) infants had no in‐bed sleepers; of the 22 (34.4%) with 1, only 3 (13.6%) were being used at the time of death. Previous work shows distribution is often inappropriate, with many low‐risk infants receiving in‐bed sleepers while high‐risk infants miss out [28]. Further research should explore how these devices are used in practice and barriers to their use when bed sharing with younger infants.

Findings from this case series highlight the urgent need to strengthen SUDI prevention messages about bed sharing. Current advice, that it is dangerous only when additional hazards are present, is no longer adequate. Guidance should distinguish intrinsic infant risk from environmental hazards and direct from indirectly bed sharing. A firmer stance is needed against direct bed sharing for vulnerable infants: those under 3 months who were exposed to smoking and/or vaping in pregnancy. For these babies, an in‐bed sleeper should be used every time bed sharing occurs. If all mothers who smoked or vaped used in‐bed sleepers instead of direct bed sharing, about 45% of SUDI deaths in this series might have been prevented, assuming sleepers offer protection comparable with separate sleep surfaces. Hazards without smoking/vaping—such as sofas, bean bags, alcohol and drugs—remain risks, but in our population account for a small proportion of deaths.

A limitation of this analysis is that it is a small case series, meaning it lacks control data for factors like vaping in pregnancy and bed sharing, preventing the calculation of odds ratios. Reliance on coronial records also meant some variables were incomplete (only 66% or 65% of the cases had full data on smoking and vaping). Nonetheless, the consistency with earlier findings supports the validity of these data [29]. We believe all 2022–2023 SUDI cases were captured, although final coroners' rulings may differ from our assessment.

## 5 Conclusion

This case series reveals that the overall mortality rate for SUDI in NZ remains unacceptably high, with increasing ethnic disparities. The most significant finding is the remarkably high proportion of deaths (72.3%) occurring while direct bed sharing, with the large majority of these (84.8%) reporting smoking and/or vaping. This combination appears to be a major driver of current SUDI rates. While prone sleeping and maternal smoking have declined among cases, vaping has emerged as a potential new or replacement risk factor requiring urgent attention. This finding highlights the urgent need for further studies examining the association of vaping with SUDI and bed sharing.

## Funding

The authors have nothing to report.

## Ethics Statement

Ethics approval for this study was granted by the NZ Northern A Health and Disability Ethics Committee (17/NTA/245).

## Conflicts of Interest

Stephanie Cowan is Director of Change for our Children, which owns the intellectual property of the Pēpi‐Pod Program and supplies it on a cost‐recovery basis; she receives a salary from this social business. The other authors declare no conflicts of interest.

## Data Availability

The data that support the findings of this study are available from Coronial Services, Ministry of Justice, New Zealand. Restrictions apply to the availability of these data, which were used under license for this study. Data are available from the author(s) with the permission of Coronial Services, Ministry of Justice, New Zealand.
